# (*5E*)-1-Benzyl-5-(3,3,3-tri­chloro-2-oxo­propyl­idene)pyrrolidin-2-one

**DOI:** 10.1107/S160053681400751X

**Published:** 2014-05-03

**Authors:** Alex Fabiani Claro Flores, Darlene Correia Flores, Juliano Rosa de Menezes Vicenti, Lucas Pizzuti, Patrick Teixeira Campos

**Affiliations:** a Escola de Química e Alimentos, Universidade Federal do Rio Grande, Av. Itália, km 08, Campus Carreiros, 96203-900, Rio Grande, RS, Brazil; b Universidade Federal da Grande Dourados, UFGD, CEP 79825-070, Dourados, MS, Brazil; c Instituto Federal Farroupilha, Campus Júlio de Castilhos, CEP 98130-000, Júlio de Castilhos, RS, Brazil

## Abstract

In the crystal structure of the title compound, C_14_H_12_Cl_3_NO_2_, no classical hydrogen-bonding inter­actions are observed. The methyl­ene fragments of the benzyl groups participate in non-classic hydrogen-bond inter­actions with the carbonyl O atoms of neighboring mol­ecules, generating co-operative centrosymmetric dimers with *R*_5_^5^(10) ring motifs. The overall mol­ecular arrangement in the unit cell seems to be highly influenced by secondary non-covalent weak C—Cl⋯π [Cl⋯*Cg*(phenyl ring) = 3.732 (2) Å] and C—O⋯π [O⋯*Cg*(pyrrolidine ring) = 2.985 (2) Å] contacts.

## Related literature   

For the synthesis of the title compound, see: Flores *et al.* (2008 ▶). For pharmacological effects, see: Van der Schyf *et al.* (2006 ▶). For non-classical weak contacts, see: Irving & Irving (1994 ▶); Bissantz *et al.* (2010 ▶). For related structures, see: Bandeira *et al.* (2013 ▶); de Oliveira *et al.* (2012 ▶); de Bittencourt *et al.* (2014 ▶).
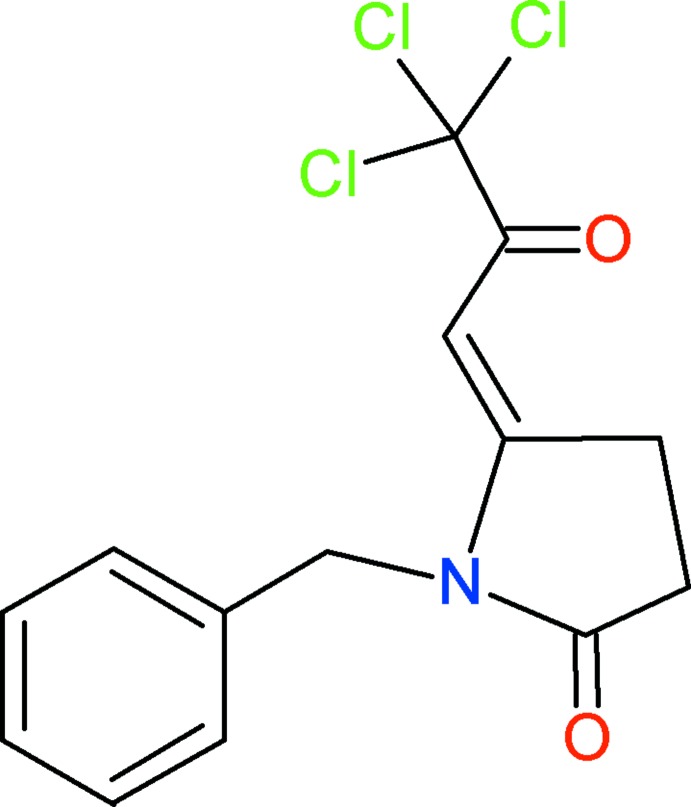


## Experimental   

### 

#### Crystal data  

C_14_H_12_Cl_3_NO_2_
*M* *_r_* = 332.60
Monoclinic, 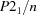

*a* = 15.7822 (5) Å
*b* = 5.8465 (2) Å
*c* = 17.4107 (5) Å
β = 105.885 (1)°
*V* = 1545.15 (8) Å^3^
*Z* = 4
Mo *K*α radiation
μ = 0.59 mm^−1^
*T* = 293 K
0.83 × 0.23 × 0.19 mm

#### Data collection  

Bruker APEXII CCD diffractometer
Absorption correction: gaussian (*XPREP*; Bruker, 2009 ▶) *T*_min_ = 0.814, *T*_max_ = 1.000
21458 measured reflections
5069 independent reflections
2436 reflections with *I* > 2σ(*I*)
*R*_int_ = 0.033

#### Refinement  

*R*[*F* ^2^ > 2σ(*F* ^2^)] = 0.054
*wR*(*F* ^2^) = 0.217
*S* = 1.00
5069 reflections
181 parameters
H-atom parameters constrained
Δρ_max_ = 0.43 e Å^−3^
Δρ_min_ = −0.51 e Å^−3^

### 

Data collection: *APEX2* (Bruker, 2009 ▶); cell refinement: *SAINT* (Bruker, 2009 ▶); data reduction: *SAINT*; program(s) used to solve structure: *SHELXS97* (Sheldrick, 2008 ▶); program(s) used to refine structure: *SHELXL97* (Sheldrick, 2008 ▶); molecular graphics: *DIAMOND* (Brandenburg, 2006 ▶); software used to prepare material for publication: *publCIF* (Westrip, 2010 ▶).

## Supplementary Material

Crystal structure: contains datablock(s) I, New_Global_Publ_Block. DOI: 10.1107/S160053681400751X/zq2220sup1.cif

Structure factors: contains datablock(s) I. DOI: 10.1107/S160053681400751X/zq2220Isup2.hkl

Supporting information file. DOI: 10.1107/S160053681400751X/zq2220Isup3.cml

CCDC reference: 740177

Additional supporting information:  crystallographic information; 3D view; checkCIF report

## Figures and Tables

**Table 1:** Hydrogen-bond geometry (Å, °)

*D*—H⋯*A*	*D*—H	H⋯*A*	*D*⋯*A*	*D*—H⋯*A*
C8—H82⋯O71^i^	0.97	2.38	3.292 (3)	156

Symmetry code: (i) 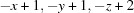.

**Fig. 1.**
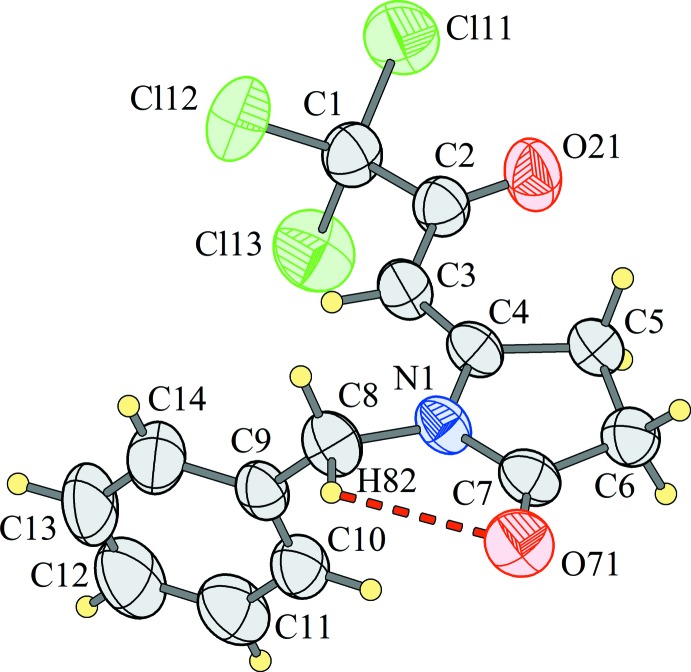
Asymmetric unit of the title compound showing an intramolecular hydrogen bond interaction represented with red dashed lines. Ellipsoid probability: 50%.

**Fig. 2.**
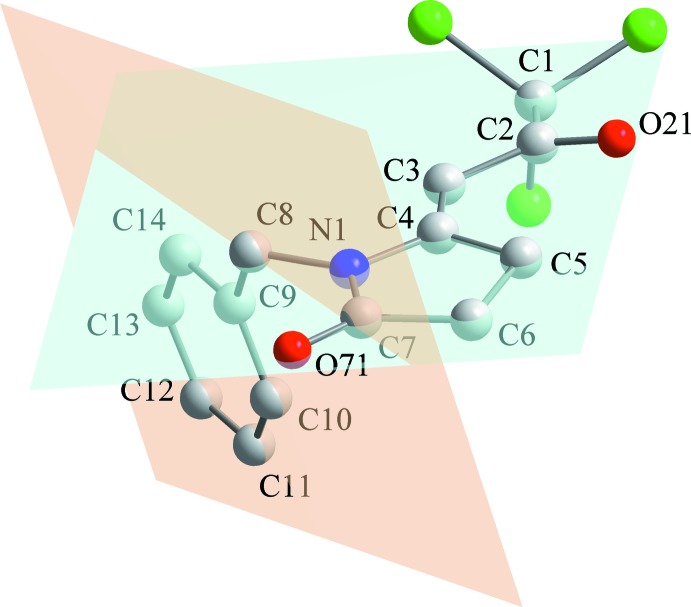
Representation of the two mean planes traced through non-hydrogenoid atoms considered for dihedral angle calculation.

**Fig. 3.**
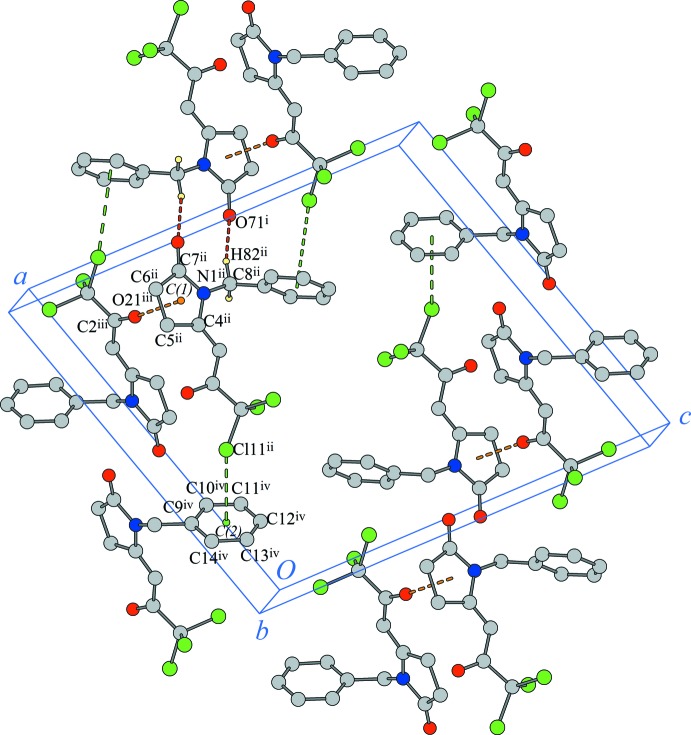
Packing diagram showing the molecular arrangement in the unit cell. Non-classic hydrogen bond interactions are represented with red dashed lines, meanwhile other weak non-covalent contacts are represented with orange and green dashed lines. Most of the hydrogen atoms were omitted for clarity. Symmetry codes: (i) –x + 3/2, y + 1/2, –z + 3/2; (ii) x + 1/2, –y + 3/2, z–1/2; (iii) –x + 1, –y + 1, –z + 1; (iv) x, y + 1, z–1.

## supplementary crystallographic information

### 1. Comment

Pyrrolidin-2-ones have received considerable attention due to their activity in *CNS* as nootropic drugs. Piracetam-like nootropics revert amnesia induced by scopolamine and other amnesing drugs, electroconvulsive shock and hypoxia with an unknown mechanism. In general, they show no affinity for the most important central receptors, but are able to modulate the action of these most important central neurotransmitters, in particular acetylcholine and glutamate. Extensive study of the modes of action of the 2-pyrrolidinones has revealed various pharmacological effects, with striking differences between drugs (Van der Schyf *et al.*, 2006). This work is a continuation of the research on the synthesis of 1-[alkyl(aryl)]-5-(3,3,3-trihalo-2-oxopropylidene)pyrrolidin-2-nes (Flores *et al.*, 2008). In the crystal structure of the title compound, C_14_H_12_O_2_NCl_3_ (Fig. 1), no classic hydrogen bonds are observed. There is a non-classic intramolecular hydrogen bond with a distance C8—H82···O71 of 2.442 Å, generating a *S*(5) ring motif (de Bittencourt *et al.*, 2014). The molecule possesses two sites revealing an interesting geometric conformation: the plane defined by the aromatic ring (r.m.s. of 0.0032 Å) revealed a dihedral angle of 87.03 (8)° with respect to the second plane formed by C1/C2/O21/C3/C4/C5/C6/C7/N1/C8/O71 atoms (r.m.s. of 0.0978 Å; Bandeira *et al.*, 2013; de Oliveira *et al.*, 2012). This almost orthogonal configuration (Fig. 2) seems to be related with the crystal packing. In this context, the CH_2_ fragment of the benzyl group participates in non-classic hydrogen bonding with carbonyl oxygen of the neighbor molecules. This feature generates co-operative centrosymmetric dimers related through inversion centers, displaying C8^ii^—H82^ii^···O71^i^ distances of 2.382 Å in a *R*_5_^5^(10) ring fashion (de Bittencourt *et al.*, 2014). The overall molecular arrangement in the unit cell (Fig. 3) seems to be highly influenced by weak interactions, where O21^iii^ are clearly pointing to *C*(1) (2.985 Å), related to the centroid of the neighbor ring formed by C4^ii^/C5^ii^/C6^ii^/C7^ii^/N1^ii^ atoms. Similarly, the centroid *C*(2) of the ring formed by C9^iv^/C10^iv^/C11^iv^/C12^iv^/C13^iv^/C14^iv^ atoms from the benzyl fragment presented directional long range interactions with adjacent Cl11^ii^ atoms (Irving & Irving, 1994; Bissantz *et al.*, 2010), with distances of 3.732 Å (symmetry codes: (i) –*x* + 3/2, *y* + 1/2, –*z* + 3/2; (ii) *x* + 1/2, –*y* + 3/2, *z*–1/2; (iii) –*x* + 1, –*y* + 1, –*z* + 1; (iv) *x*, *y* + 1, *z*–1).

### 2. Experimental

To a stirred solution of methyl 7,7,7-trichloro-4-methoxy-6-oxo-3-heptenoate (5 mmol, 1.52 g) in CHCl_3_ (5 ml) kept at 25 °C, was added benzylamine (Aldrich, 185701, 5.1 mmol, 0.58 ml) in CHCl_3_ (5 ml). The mixture was stirred at 25 °C for 2 h. Then the solvent was evaporated and residue was dried under vacuum. The brown amorphous solid was recrystallized in hexane to furnish yellowish needles with 94% yield. *M*.p. 88 - 89°C. ^1^H NMR (400 MHz, CDCl_3_/TMS): δ 2.72 (m, 2H, H3), 3.34 (m, 2H, H4), 4.8 (s, 2H, H9), 6.2 (s, 1H, H6), 7.3 (m, 5H, Ph) p.p.m. ^13^C NMR (100 MHz, CDCl_3_): 26.1, 27.5, 44.6, 91.7, 97.1, 127.6, 128.1, 128.9, 134.1, 166.4, 177.1, 180 p.p.m. Crystals were grown from a diluted hexane solution at room temperature.

### 3. Refinement

All H atoms attached to C atoms were positioned with idealized geometry and were refined isotropic with *U*_eq_(H) set to 1.2 times of the *U*_eq_(C). It was used a riding model with C—H = 0.97 Å for CH_2_ and C—H = 0.93 Å for CH. Reflection (101) was omitted due to the large difference observed between *F_o_*^2^ and Fc^2^. The crystals were relatively weak in terms of intensity of diffraction, prompting us to select a big crystal for the measurement and consequently a collimator with a larger diameter (0.6 mm) than in routine studies.

### Crystal data

**Table:**

C_14_H_12_Cl_3_NO_2_	*F*(000) = 680
*M**_r_* = 332.60	*D*_x_ = 1.430 Mg m^−^^3^
Monoclinic, *P*2_1_/*n*	Melting point: 361 K
Hall symbol: -P 2yn	Mo *K*α radiation, λ = 0.71073 Å
*a* = 15.7822 (5) Å	Cell parameters from 3113 reflections
*b* = 5.8465 (2) Å	θ = 2.4–21.5°
*c* = 17.4107 (5) Å	µ = 0.59 mm^−^^1^
β = 105.885 (1)°	*T* = 293 K
*V* = 1545.15 (8) Å^3^	Block, yellow
*Z* = 4	0.83 × 0.23 × 0.19 mm

### Data collection

**Table:**

Bruker APEXII CCD diffractometer	5069 independent reflections
Radiation source: fine-focus sealed tube	2436 reflections with *I* > 2σ(*I*)
Graphite monochromator	*R*_int_ = 0.033
*φ* and *ω* scans	θ_max_ = 31.5°, θ_min_ = 2.7°
Absorption correction: gaussian (*XPREP*; Bruker, 2009)	*h* = −23→23
*T*_min_ = 0.814, *T*_max_ = 1	*k* = −8→3
21458 measured reflections	*l* = −25→25

### Refinement

**Table:**

Refinement on *F*^2^	Primary atom site location: structure-invariant direct methods
Least-squares matrix: full	Secondary atom site location: difference Fourier map
*R*[*F*^2^ > 2σ(*F*^2^)] = 0.054	Hydrogen site location: inferred from neighbouring sites
*wR*(*F*^2^) = 0.217	H-atom parameters constrained
*S* = 1.00	*w* = 1/[σ^2^(*F*_o_^2^) + (0.1095*P*)^2^ + 0.2217*P*] where *P* = (*F*_o_^2^ + 2*F*_c_^2^)/3
5069 reflections	(Δ/σ)_max_ < 0.001
181 parameters	Δρ_max_ = 0.43 e Å^−^^3^
0 restraints	Δρ_min_ = −0.51 e Å^−^^3^

### Special details

**Table:**

Experimental. Gaussian absorption correction based on the face-indexed crystal size
Geometry. All e.s.d.'s (except the e.s.d. in the dihedral angle between two l.s. planes) are estimated using the full covariance matrix. The cell e.s.d.'s are taken into account individually in the estimation of e.s.d.'s in distances, angles and torsion angles; correlations between e.s.d.'s in cell parameters are only used when they are defined by crystal symmetry. An approximate (isotropic) treatment of cell e.s.d.'s is used for estimating e.s.d.'s involving l.s. planes.
Refinement. Refinement of *F*^2^ against ALL reflections. The weighted *R*-factor *wR* and goodness of fit *S* are based on *F*^2^, conventional *R*-factors *R* are based on *F*, with *F* set to zero for negative *F*^2^. The threshold expression of *F*^2^ > σ(*F*^2^) is used only for calculating *R*-factors(gt) *etc*. and is not relevant to the choice of reflections for refinement. *R*-factors based on *F*^2^ are statistically about twice as large as those based on *F*, and *R*- factors based on ALL data will be even larger.

### Fractional atomic coordinates and isotropic or equivalent isotropic displacement parameters (Å^2^)

**Table:**

	*x*	*y*	*z*	*U*_iso_*/*U*_eq_	
Cl11	−0.04344 (5)	−0.0759 (2)	0.73859 (6)	0.0958 (3)	
Cl12	0.01375 (6)	0.32178 (15)	0.83385 (6)	0.0921 (3)	
Cl13	0.03185 (7)	−0.11995 (17)	0.90926 (6)	0.0955 (3)	
O71	0.50426 (12)	0.2290 (4)	0.95046 (11)	0.0688 (5)	
N1	0.35352 (12)	0.2022 (3)	0.92101 (10)	0.0485 (5)	
O21	0.13827 (13)	−0.1294 (4)	0.75824 (13)	0.0766 (6)	
C9	0.30299 (15)	0.3271 (4)	1.03807 (14)	0.0517 (6)	
C4	0.28593 (15)	0.0825 (4)	0.87105 (13)	0.0473 (5)	
C2	0.13065 (16)	−0.0046 (4)	0.81150 (14)	0.0541 (6)	
C3	0.20017 (17)	0.1159 (4)	0.86770 (14)	0.0537 (6)	
H3	0.1858	0.2195	0.9027	0.064*	
C8	0.34165 (19)	0.3933 (4)	0.97121 (15)	0.0573 (6)	
H81	0.3035	0.5058	0.9379	0.069*	
H82	0.3984	0.4653	0.9939	0.069*	
C6	0.42339 (17)	−0.0584 (5)	0.85719 (15)	0.0584 (6)	
H62	0.4489	−0.1974	0.8843	0.070*	
H61	0.4507	−0.0263	0.8148	0.070*	
C5	0.32443 (16)	−0.0833 (4)	0.82383 (14)	0.0507 (5)	
H51	0.3062	−0.0455	0.7674	0.061*	
H52	0.3060	−0.2384	0.8308	0.061*	
C10	0.32564 (18)	0.1269 (5)	1.08009 (16)	0.0614 (7)	
H10	0.3654	0.0275	1.0667	0.074*	
C7	0.43644 (17)	0.1369 (4)	0.91455 (14)	0.0536 (6)	
C11	0.2900 (2)	0.0712 (7)	1.14227 (18)	0.0810 (9)	
H11	0.3059	−0.0653	1.1699	0.097*	
C14	0.2441 (2)	0.4725 (6)	1.0591 (2)	0.0778 (8)	
H14	0.2273	0.6087	1.0315	0.093*	
C13	0.2094 (2)	0.4110 (8)	1.1238 (2)	0.0972 (12)	
H13	0.1704	0.5088	1.1389	0.117*	
C1	0.03650 (17)	0.0294 (5)	0.82250 (16)	0.0615 (6)	
C12	0.2326 (2)	0.2126 (8)	1.1629 (2)	0.0930 (11)	
H12	0.2089	0.1729	1.2044	0.112*	

### Atomic displacement parameters (Å^2^)

**Table:**

	*U*^11^	*U*^22^	*U*^33^	*U*^12^	*U*^13^	*U*^23^
Cl11	0.0520 (4)	0.1369 (8)	0.0932 (6)	−0.0116 (4)	0.0110 (4)	−0.0291 (5)
Cl12	0.0838 (6)	0.0785 (6)	0.1221 (7)	0.0163 (4)	0.0416 (5)	−0.0080 (5)
Cl13	0.0997 (7)	0.1099 (7)	0.0892 (6)	−0.0161 (5)	0.0465 (5)	0.0130 (5)
O71	0.0531 (10)	0.0804 (13)	0.0728 (11)	−0.0168 (9)	0.0169 (9)	−0.0078 (10)
N1	0.0497 (10)	0.0571 (11)	0.0417 (9)	−0.0128 (8)	0.0175 (8)	−0.0064 (8)
O21	0.0558 (11)	0.0922 (15)	0.0809 (13)	−0.0063 (10)	0.0169 (10)	−0.0388 (11)
C9	0.0454 (12)	0.0599 (14)	0.0497 (12)	−0.0087 (10)	0.0130 (10)	−0.0156 (11)
C4	0.0522 (13)	0.0517 (12)	0.0396 (11)	−0.0085 (10)	0.0155 (9)	−0.0008 (9)
C2	0.0508 (13)	0.0580 (14)	0.0547 (14)	−0.0029 (11)	0.0166 (11)	−0.0057 (11)
C3	0.0545 (13)	0.0597 (14)	0.0491 (13)	−0.0055 (11)	0.0176 (11)	−0.0090 (10)
C8	0.0633 (15)	0.0533 (13)	0.0576 (14)	−0.0138 (11)	0.0205 (12)	−0.0080 (11)
C6	0.0532 (14)	0.0696 (16)	0.0537 (14)	0.0010 (11)	0.0167 (11)	−0.0030 (12)
C5	0.0551 (13)	0.0542 (13)	0.0441 (11)	−0.0039 (10)	0.0158 (10)	−0.0027 (10)
C10	0.0568 (15)	0.0748 (17)	0.0579 (14)	−0.0009 (12)	0.0245 (12)	−0.0040 (13)
C7	0.0545 (14)	0.0640 (14)	0.0433 (12)	−0.0096 (11)	0.0150 (10)	0.0011 (10)
C11	0.088 (2)	0.101 (2)	0.0599 (17)	−0.0077 (18)	0.0312 (16)	0.0037 (16)
C14	0.0735 (19)	0.081 (2)	0.082 (2)	0.0109 (15)	0.0257 (16)	−0.0153 (16)
C13	0.075 (2)	0.140 (4)	0.089 (2)	0.013 (2)	0.0438 (19)	−0.033 (2)
C1	0.0522 (14)	0.0691 (16)	0.0661 (16)	−0.0036 (12)	0.0208 (12)	−0.0052 (13)
C12	0.088 (2)	0.131 (3)	0.074 (2)	−0.013 (2)	0.0459 (19)	−0.012 (2)

### Geometric parameters (Å, º)

**Table:**

Cl11—C1	1.760 (3)	C8—H82	0.9700
Cl12—C1	1.769 (3)	C6—C7	1.494 (3)
Cl13—C1	1.764 (3)	C6—C5	1.517 (4)
O71—C7	1.208 (3)	C6—H62	0.9700
N1—C4	1.370 (3)	C6—H61	0.9700
N1—C7	1.397 (3)	C5—H51	0.9700
N1—C8	1.462 (3)	C5—H52	0.9700
O21—C2	1.212 (3)	C10—C11	1.389 (4)
C9—C10	1.375 (4)	C10—H10	0.9300
C9—C14	1.381 (4)	C11—C12	1.345 (5)
C9—C8	1.505 (3)	C11—H11	0.9300
C4—C3	1.353 (3)	C14—C13	1.426 (5)
C4—C5	1.503 (3)	C14—H14	0.9300
C2—C3	1.439 (4)	C13—C12	1.345 (6)
C2—C1	1.562 (3)	C13—H13	0.9300
C3—H3	0.9300	C12—H12	0.9300
C8—H81	0.9700		
			
C4—N1—C7	113.14 (19)	C6—C5—H51	110.8
C4—N1—C8	124.4 (2)	C4—C5—H52	110.8
C7—N1—C8	122.2 (2)	C6—C5—H52	110.8
C10—C9—C14	118.6 (2)	H51—C5—H52	108.8
C10—C9—C8	121.9 (2)	C9—C10—C11	120.8 (3)
C14—C9—C8	119.4 (3)	C9—C10—H10	119.6
C3—C4—N1	123.4 (2)	C11—C10—H10	119.6
C3—C4—C5	128.2 (2)	O71—C7—N1	123.6 (2)
N1—C4—C5	108.4 (2)	O71—C7—C6	128.8 (2)
O21—C2—C3	126.8 (2)	N1—C7—C6	107.6 (2)
O21—C2—C1	117.9 (2)	C12—C11—C10	120.7 (4)
C3—C2—C1	115.4 (2)	C12—C11—H11	119.7
C4—C3—C2	121.8 (2)	C10—C11—H11	119.7
C4—C3—H3	119.1	C9—C14—C13	119.1 (3)
C2—C3—H3	119.1	C9—C14—H14	120.5
N1—C8—C9	114.3 (2)	C13—C14—H14	120.5
N1—C8—H81	108.7	C12—C13—C14	120.5 (3)
C9—C8—H81	108.7	C12—C13—H13	119.8
N1—C8—H82	108.7	C14—C13—H13	119.8
C9—C8—H82	108.7	C2—C1—Cl11	110.10 (18)
H81—C8—H82	107.6	C2—C1—Cl13	107.82 (19)
C7—C6—C5	105.6 (2)	Cl11—C1—Cl13	110.45 (15)
C7—C6—H62	110.6	C2—C1—Cl12	111.45 (18)
C5—C6—H62	110.6	Cl11—C1—Cl12	108.04 (16)
C7—C6—H61	110.6	Cl13—C1—Cl12	109.00 (15)
C5—C6—H61	110.6	C13—C12—C11	120.3 (3)
H62—C6—H61	108.8	C13—C12—H12	119.8
C4—C5—C6	104.88 (19)	C11—C12—H12	119.8
C4—C5—H51	110.8		
			
C7—N1—C4—C3	179.4 (2)	C8—N1—C7—O71	1.3 (4)
C8—N1—C4—C3	−5.8 (3)	C4—N1—C7—C6	−3.7 (3)
C7—N1—C4—C5	−0.5 (3)	C8—N1—C7—C6	−178.6 (2)
C8—N1—C4—C5	174.3 (2)	C5—C6—C7—O71	−173.7 (3)
N1—C4—C3—C2	176.7 (2)	C5—C6—C7—N1	6.2 (3)
C5—C4—C3—C2	−3.4 (4)	C9—C10—C11—C12	−0.2 (5)
O21—C2—C3—C4	−6.5 (4)	C10—C9—C14—C13	0.4 (4)
C1—C2—C3—C4	172.5 (2)	C8—C9—C14—C13	−178.5 (3)
C4—N1—C8—C9	67.9 (3)	C9—C14—C13—C12	−1.0 (6)
C7—N1—C8—C9	−117.8 (2)	O21—C2—C1—Cl11	−13.2 (3)
C10—C9—C8—N1	38.3 (3)	C3—C2—C1—Cl11	167.62 (19)
C14—C9—C8—N1	−142.8 (3)	O21—C2—C1—Cl13	107.3 (3)
C3—C4—C5—C6	−175.6 (2)	C3—C2—C1—Cl13	−71.8 (3)
N1—C4—C5—C6	4.3 (2)	O21—C2—C1—Cl12	−133.1 (2)
C7—C6—C5—C4	−6.3 (2)	C3—C2—C1—Cl12	47.8 (3)
C14—C9—C10—C11	0.2 (4)	C14—C13—C12—C11	1.0 (6)
C8—C9—C10—C11	179.1 (3)	C10—C11—C12—C13	−0.4 (6)
C4—N1—C7—O71	176.2 (2)		

### Hydrogen-bond geometry (Å, º)

**Table:**

*D*—H···*A*	*D*—H	H···*A*	*D*···*A*	*D*—H···*A*
C8—H82···O71^i^	0.97	2.38	3.292 (3)	156

Symmetry code: (i) −*x*+1, −*y*+1, −*z*+2.
